# High Seroprevalence Rates of *Toxoplasma gondii* and *Neospora caninum* in Dogs in the Pantanal Region of Mato Grosso, Brazil

**DOI:** 10.1007/s11686-026-01241-0

**Published:** 2026-03-17

**Authors:** Gabriel Lucas Artiaga-Silva, Álvaro Felipe de Lima Ruy Dias, Matheus Roberto Carvalho, Kamilla Silva Melo, Arleana do Bom Parto Ferreira de Almeida, João Luis Garcia, Richard de Campos Pacheco, Valéria Régia Franco Sousa

**Affiliations:** 1 Faculty of Veterinary Medicine, Federal University of Mato Grosso, Cuiabá, MT Brazil; 2 National Institute for Research on Human and Animal Toxoplasmosis (INCT), State University of Londrina, Londrina, PR Brazil

**Keywords:** Coinfection, Risk factors, *Leishmania infantum*, Prevalence

## Abstract

**Purpose:**

*Toxoplasma gondii* and *Neospora caninum* are protozoan that infect animals worldwide. Dogs act as sentinels of these infections, indicating potential risks to human health, particularly in the case of *T. gondii* due to its zoonotic nature. Therefore, we aimed to determine the seroprevalence and associated factors for *T. gondii* and *N. caninum* infections in dogs living in the Pantanal biome, Brazil.

**Methods:**

This study evaluated the seroprevalence in 743 serum samples from dogs in Barão de Melgaço and Nossa Senhora do Livramento, municipalities endemic for canine visceral leishmaniasis located in the Pantanal biome of Mato Grosso, Brazil, by Indirect Immunofluorescence Assay.

**Results:**

Overall, 66.4% (*n* = 493) were positive for *T. gondii* and 12.4% (*n* = 92) for *N. caninum*. Seropositivity was associated with dogs older than 3 years (*T. gondii*—*p* = 0.001; OR = 2.579; *N. caninum*—*p* = 0.004; OR = 7.621), living together with other dogs and/or cats (*T. gondii*—*p* = 0.04; *N. caninum*—*p* = 0.005), and absence of a public sewage system at home (*T. gondii*—*p* = 0.044; OR = 4.730; *N. caninum*—*p* = 0.035; OR = 1.376). For *T. gondii*, additional associations were found with street access (*p* = 0.015; OR = 3.966), contact with rodents (*p* = 0.025; OR = 1.539), and a diet including leftover food (*p* = 0.025; OR = 2.405). Infection by *N. caninum* was more frequent in rural dogs (*p* = 0.011; OR = 4.857) and in those coinfected with *Leishmania infantum* (*p* < 0.001; OR = 6.407).

**Conclusion:**

The investigated regions are endemic for *T. gondii* and *N. caninum*, with increased risk associated with environmental conditions and dog management practices. Furthermore, dogs infected with *L. infantum* showed a higher likelihood of coinfection with *N. caninum*.

## Introduction

In the One Health context, parasitic diseases are important research targets because they are directly associated with socioeconomic vulnerability and inadequate living conditions [1]. Examples include toxoplasmosis, neosporosis, and visceral leishmaniasis, which can cause severe clinical disorders in dogs [2, 3] and lead to significant economic losses [4–6].

In dogs, toxoplasmosis, caused by the obligate intracellular protozoan *Toxoplasma gondii* [3], shows high prevalence rates that can reach up to 70% in Brazil. Although felids are the definitive hosts, dogs may contribute to the mechanical dissemination of the parasite [7, 8]. Considering the parasite can infect a wide range of mammals and birds [7, 9] as well as some reptile species [10], dogs have epidemiological importance as sentinels, and the identification of seropositive animals may indicate a potential risk of infection for humans [11].

*Neospora caninum*, another obligate intracellular protozoan, can infect canids, which serve as definitive hosts, as well as various herbivores, which act as intermediate hosts [12]. Infection can cause neurological disease in dogs and is one of the main causes of abortion in cattle herds [9]. Thus, dogs, particularly those living in rural areas, play a key role in the epidemiology of the disease, significantly contributing to the circulation of the parasite in the environment and increasing the risk of infection for intermediate hosts, such as cattle and wildlife [13, 14].

The Pantanal is a biome characterized by high biodiversity and intense interactions among humans, domestic animals, and wildlife [15, 16]. In addition, it is considered an endemic area for canine visceral leishmaniasis due to the distribuition of *Lutzomyia longipalpis*, the vector of *Leishmania infantum* [17]. In this context, the aim of this study was to determine the prevalence and associated risk factors for *T. gondii* and *N. caninum* infections by assessing the presence of antibodies in dogs living in areas endemic for canine visceral leishmaniasis in the Pantanal biome of Mato Grosso, Brazil.

## Materials and Methods

### Study Area and Sample Size Calculation

**Fig. 1:**
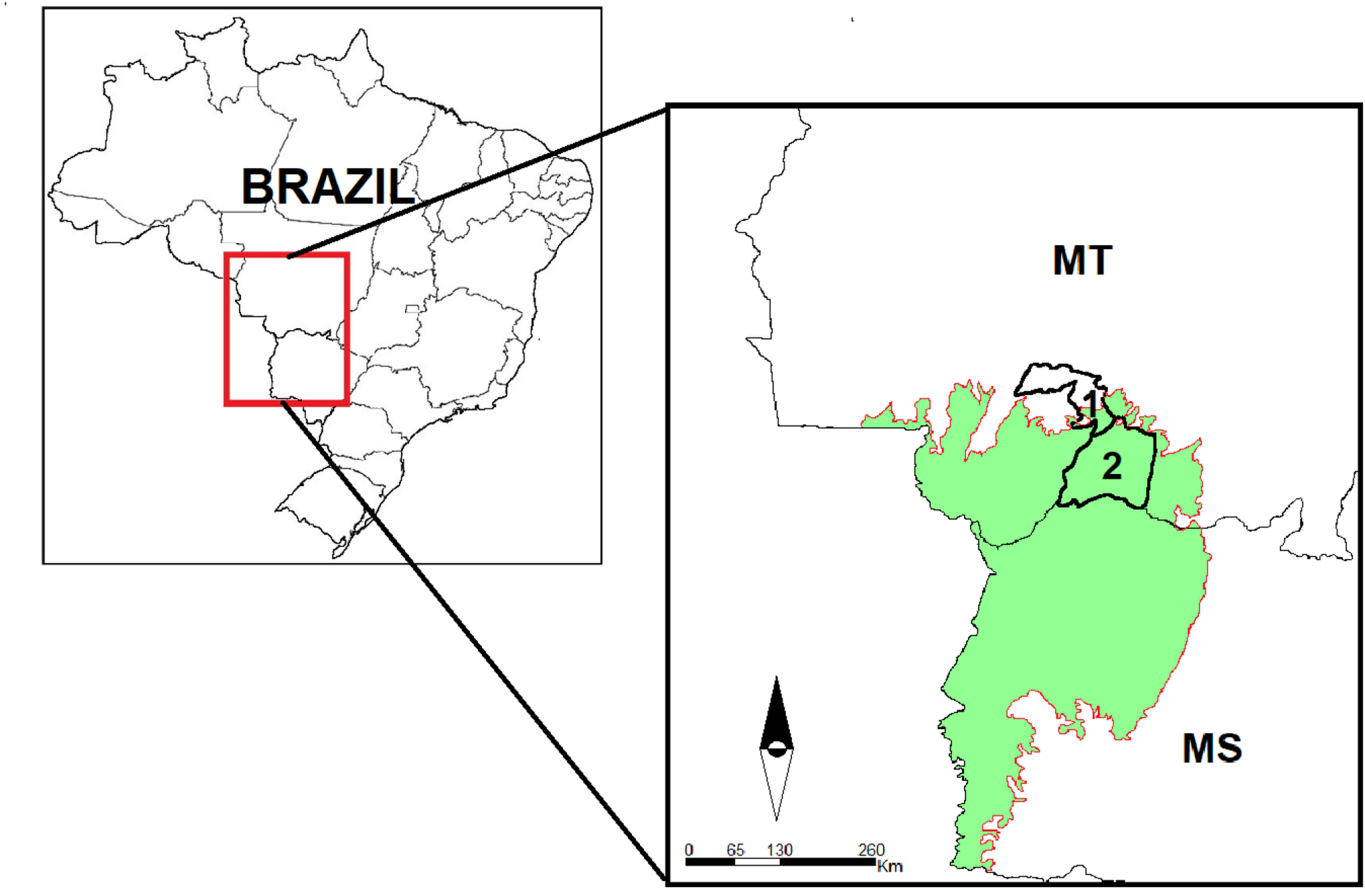
Boundaries of the municipalities of Nossa Senhora do Livramento (1) and Barão de Melgaço (2) in the Pantanal biome (green) located in the states of Mato Grosso (MT) and Mato Grosso do Sul (MS)—Brazil

In this study, 743 canine serum samples were analyzed from two municipalities located in the northern Pantanal region of Mato Grosso, Brazil: 402 samples from Barão de Melgaço (16°11′40″S and 55°58′03″W) (Fig. 1) and 341 samples from Nossa Senhora do Livramento (15°46′30″S and 56°20′44″W) (Fig. 1), collected between 2015 and 2018. Sample size calculations were based on an expected prevalence of 50%, a 95% confidence interval, and a 5% margin of error, estimating the canine population using a dog-to-human ratio of 1:7 [18, 19].

### Antibody Detection

Antibody detection was performed using the Indirect Immunofluorescence Assay (IFA) employing a canine anti-IgG conjugate obtained from rabbit inoculations and labeled with fluorescein isothiocyanate (FITC–Sigma^®^). Slides for *T. gondii* were prepared using RH strains, with a cut off titer of 1:16 [20, 21]. For *N. caninum*, the cutoff was 1:50 [20], using NC-1 strains as antigens in slide preparation. Each slide included negative and positive serum controls. Reactions were considered positive when tachyzoites exhibited complete peripheral fluorescence [21]. All samples had previously been evaluated for *L. infantum* seropositivity using a rapid immunochromatographic test (Dual Path Platform–DPP, Biomanguinhos®, Brazil) and enzyme-linked immunosorbent assay (ELISA, Biomanguinhos®, Brazil) [18, 19].

### Epidemiological Information

The following information was collected for the entire dog population: breed (defined; undefined), sex (male; female), age (puppy—up to 12 months; young—1 to 3 years; adult—over 3 years) [22], type of diet (commercial feed; leftover food; raw meat; mixed), type of household sewage system (public; open-air; septic tank), street access, cohabitation with other dogs and/or cats, property location (rural; urban), and the presence of rodents and/or marsupials in the dog’s accessible environment.

### Statistical Analysis

These data were analyzed to determine risk factors by assessing the association between variables and infection rates of *T. gondii* and *N. caninum* using the chi-square test and two-step logistic regression, including univariate and multivariate analyses, performed with Jamovi software (2.3.28). Variables with *p* ≤ 0.20 in the univariate chi-square analysis were included in the multivariate analysis, where *p* ≤ 0.05 was considered statistically significant.

## Results

Among the 743 canine serum samples tested by Indirect Immunofluorescence Assay, antibodies against *T. gondii* were detected in 493 samples (*n* = 493; 66.4%) (95% CI 62.9–69.7%) at a 1:16 dilution, while antibodies against *N. caninum* were detected in 92 samples (*n* = 92; 12.4%) (95% CI 10.0–14.7%) at a 1:50 dilution.

In the statistical analysis (Table 1), no association (*p* > 0.05) was found between the sex of the dogs and the detection of antibodies against *T. gondii* and *N. caninum*. The occurrence of antibodies against both agents was more prevalent in adult dogs (*T. gondii* – *p* = 0.001; OR = 2.579; *N. caninum* – *p* = 0.004; OR = 7.621). Regarding breed, purebred dogs were more seropositive to *N. caninum* (*p* = 0.013; OR = 3.16). The breeds identified among the seropositive dogs to *N. caninum* included Boxer (*n* = 3), Pinscher (*n* = 2), as well as other breeds such as Bulldog, American Pit Bull Terrier and Fila Brasileiro.

Seropositivity to *T. gondii* and *N. caninum*, separately, was associated with the absence of a public sanitary sewage system in the household where the dog lived (*T. gondii*—*p* = 0.044; OR = 4.730; *N. caninum*—*p* = 0.035; OR = 1.376) and with contact with other domestic animals (dogs and/or cats) (*T. gondii*—*p* = 0.037;OR = 1.180; *N. caninum*—*p* = 0.005; OR = 1.513). An association was also found between the occurrence of antibodies against *T. gondii* and dogs fed with leftover food (*p* = 0.025; OR = 2.405), those with access to the street (*p* = 0.030; OR = 1.558), and those living in environments where rodents and/or marsupials were seen (*p* = 0.025; OR = 1.539). Additionally, infection with *N. caninum* was associated with living in rural areas (*p* = 0.011; OR = 4.857).

**Fig. 2:**
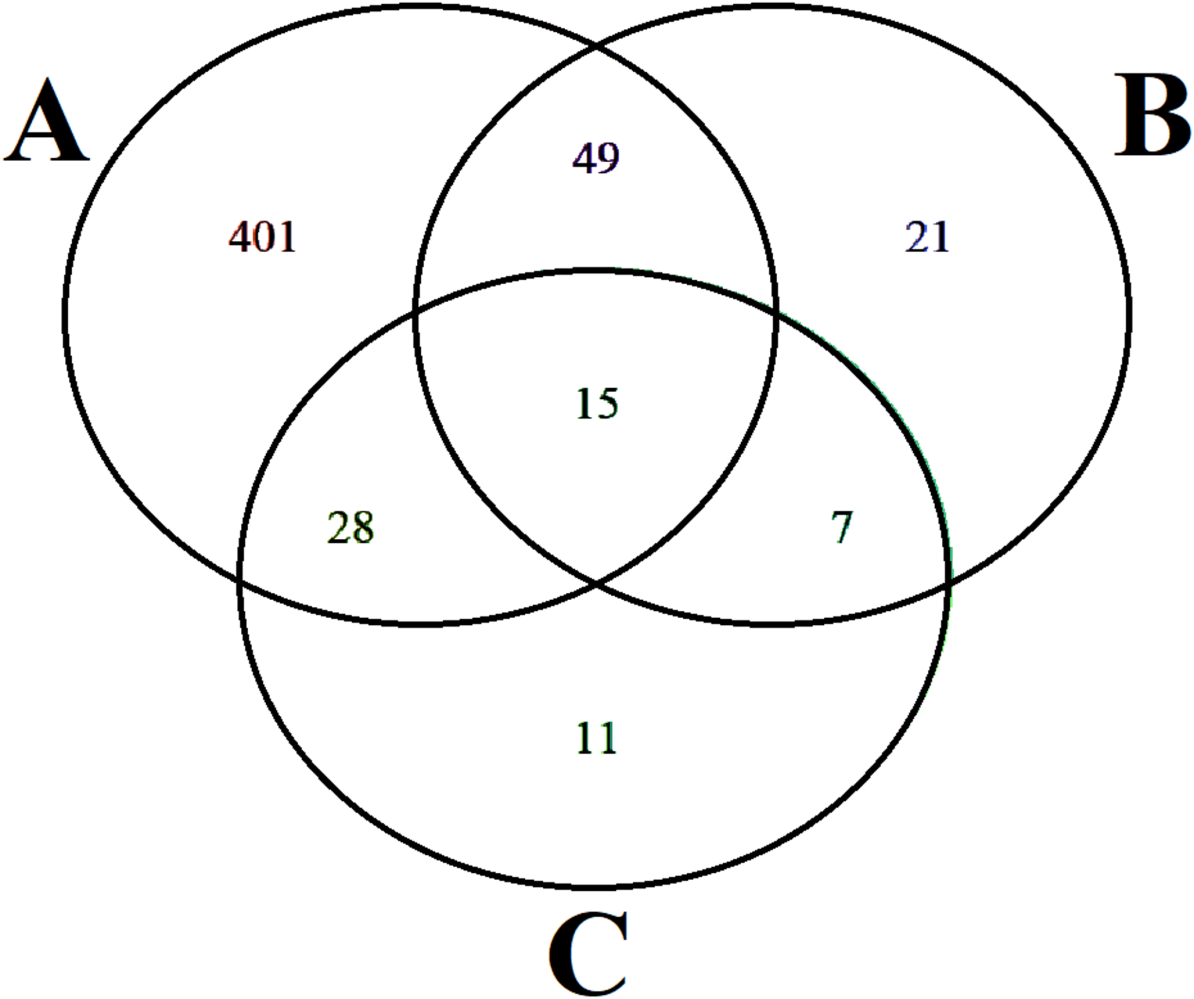
Venn diagram representing the total number of seropositive dogs for *Toxoplasma gondii* (**A**), *Neospora caninum* (**B**), and *Leishmania infantum* (**C**)

In the analysis of coinfections (Fig. 2), coinfection with *T. gondii* and *L. infantum* was observed in 5.8% of the evaluated dogs (43/743), with no statistically significant association detected between these infections (*p* > 0.05). Similarly, coinfection with *T. gondii* and *N. caninum* was identified in 8.6% of the dogs (64/743), also showing no statistically significant association (*p* > 0.05). In contrast, coinfection with *N. caninum* and *L. infantum* was observed in 2.9% of the dogs (22/743), presenting a statistically significant association (*p* < 0.001; OR = 6.407).

**Table 1:** Multivariate analysis of general and environmental characteristics associated with *Toxoplasma gondii* and *Neospora caninum* infection in dogs from municipalities in the Pantanal region of Mato Grosso, Brazil

Variable	Category (n)	*Toxoplasma gondii*		*Neospora caninum*	
		Seropositive (%)	p (OR*)	Seropositive (%)	p (OR*)
Breed	Purebred (54)	32 (59.3)	0.797	12 (22.2)	0.013 (3.167)
	Mixed breed (689)	461 (66.9)		80 (11.6)	
Sex	Male (435)	291 (66.9)	0.907	56 (12.9)	0.824
	Female (308)	202 (65.6)		36 (11.7)	
Age	Puppy (95)	43 (45.3)	0.001 (2.579)	2 (2.1)	0.004 (7.621)
	Young (229)	157 (68.6)		25 (10.9)	
	Adult (419)	293 (69.9)		65 (15.5)	
Diet	Commercial feed (46)	24 (52.2)	0.025 (2.405)	3 (6.5)	0.426
	Table scraps (135)	104 (77.0)		13 (9.6)	
	Raw meat (7)	5 (71.4)		1 (14.3)	
	Mixed diet (555)	360 (64.9)		75 (13.5)	
Contact with other animals	None (166)	108 (65.1)	0.037 (1.180)	16 (9.6)	0.005 (1.513)
	Dog (327)	210 (64.2)		40 (12.2)	
	Cat (30)	25 (83.3)		0	
	Both (220)	150 (68.2)		36 (16.4)	
Rodents/marsupials	No (281)	171 (60.9)	0.025 (1.539)	30 (10.7)	0.581
	Yes (462)	322 (69.7)		62 (13.4)	
Access to the street	No access (220)	128 (58.2)	0.030 (1.558)	32 (14.5)	0.138
	With access (523)	365 (69.8)		60 (11.5)	
Type of sewage system	Public sewer system (69)	38 (55.1)	0.044 (4.730)	6 (8.7)	0.035 (1.376)
	Open sewage (15)	12 (80.0)		0	
	Septic tank (659)	443 (67.2)		86 (13.1)	
Place of residence	Rural area (336)	225 (67.0)	0.499	50 (14.9)	0.011 (4.857)
	Urban area (407)	268 (65.8)		42 (10.3)	
Municipality	Barão de Melgaço (402)	270 (67.2)	0.718	51 (12.7)	0.817
	Nossa Senhora do Livramento (341)	223 (65.4)		41 (12.0)	
Serology for *Leishmania* spp.	Seronegative (682)	450 (66.0)	0.802	70 (10.3)	< 0 0.001 (6.407)
	Seropositive (61)	43 (70.5)		22 (36.1)	

*OR: Odds ratio

## Discussion

The seroprevalence of antibodies against *T. gondii* (66.4%) and *N. caninum* (12.4%) observed in this study highlights the extensive circulation of these protozoa among domestic dogs in the Pantanal biome of Mato Grosso, emphasizing the epidemiological significance of these infections compared to global prevalence rates [7, 12], which can reach up to 98% for *T. gondii* [23] and an average prevalence of 17.14% for *N. caninum* [12]. In Brazil, other studies reported slightly higher rates than those found here, with up to 70% of dogs seropositive for *T. gondii* [7] and an average of 13.72% for *N. caninum* [12]. Differences in prevalence between studies may be attributed to varying environmental conditions, differences in population sizes, the serological cutoff values applied, and the use of diagnostic methods with differing sensitivities and specificities [7].

The study municipalities, Barão de Melgaço and Nossa Senhora do Livramento, are located within the Pantanal biome. The Pantanal is recognized for its vast biodiversity, hosting a wide variety of wildlife species [24], and it is also considered an important region for Brazilian livestock production [25]. Studies conducted in other areas of Mato Grosso that are also part of the Pantanal biome reinforce this dynamic. Assessing riverside communities along the Cuiabá River, researchers found *T. gondii* prevalence ranging from 25.6 to 64.3% in dogs [26]. For *N. caninum*, a prevalence of 21.56% was reported in dogs from the municipality of Poconé [27], indicating that different regions of the Pantanal exhibit varying levels of exposure to these agents.

In the context of this study, the prevalence of antibodies against *T. gondii* in dogs was significantly higher than that of *N. caninum*. This difference may be explained by the diversity of wild felid species in the biome, whose shed oocysts contribute to environmental contamination and to the infection of intermediate hosts [28, 29]. This finding also reinforces the importance of the parasite as a human health threat, since people share the same environment as dogs, and studies have found identical strains infecting both species [30]. Data from the Ministry of Health show that, between 2019 and 2024, ten cases of gestational toxoplasmosis were reported in the municipalities of Barão de Melgaço and Nossa Senhora do Livramento [31], highlighting the need for continuous surveillance, especially in areas where *T. gondii* circulation is high. Dogs, considered sentinel animals, play a crucial role in the dynamics of these parasitic diseases [11]. Conversely, neosporosis is particularly relevant due to cattle farming, as cattle are important intermediate hosts [32].

Prevalence studies are essential for understanding the epidemiological dynamics in the region, allowing the identification of circulation patterns of these agents and the potential risks to animal and human health, as well as supporting the planning of control, monitoring, and sanitary management measures [11, 33], ultimately reducing impacts on wildlife [29, 34] and on the economy [4, 5]. Likewise, the analysis of risk factors for *T. gondii* and *N. caninum* infections help to better understand their epidemiology and provides information that supports the development of action plans aimed at controlling and preventing these infections [33, 34].

The occurrence of antibodies against *T. gondii* and *N. caninum* was similar between male and female dogs, consistent with other studies [35–38], indicating that both sexes are equally susceptible to infection by these protozoa. However, some studies reported a higher likelihood of *T. gondii* infection in male dogs [22], while others found *N. caninum* to be more prevalent in female dogs [39]. Regarding age-related risk, it was observed that antibody rates for both agents increased as dogs aged. Though, this increase was statistically significant only when comparing puppies (up to 12 months) with adult dogs. Although age-related prevalence variation has also been reported in other studies [35–39], the differences were not always statistically significant. Nonetheless, these data indicate that the likelihood of infection tends to increase with age due to prolonged exposure [22]. In addition, purebred dogs showed a statistically significant association with *N. caninum* infection, differing from reports by other authors who identified a higher risk among mixed-breed dogs [40]. However, this divergence should be interpreted with caution, as purebred dogs were underrepresented in the sampling of the present study.

In dogs, *T. gondii* and *N. caninum* infections can also be associated with lifestyle factors, including dietary habits such as the consumption of raw meat [13], living together with other dogs or cats [8, 35], yard access, and coprophagy [8]. In the population of this study, living together with other dogs and/or cats, compared to dogs without contact with other animals, suggested an increased risk of infection for both agents. Similarly, dogs living in households with a public sewage system had a lower likelihood of infection with *N. caninum* and *T. gondii*, indicating that poor sanitary conditions may favor the dissemination of these agents, which can be present in wastewater [4, 41].

Dogs with street access, presence of rodents or marsupials in the household, and those fed of leftover food showed a higher risk of *T. gondii* infection. This finding is consistent with Brasil et al. [42], who also reported higher prevalence in dogs that roamed the streets, and with Ijaz et al. [43], who emphasize the role of rodents as intermediate hosts in transmission, since predation allows direct ingestion of tissue cysts present in their muscles and viscera. In this study, the diet including leftover food was identified as a risk factor for *T. gondii* infection, but no association was found with the ingestion of raw meat, other risk factor reported by Mascolli et al. [44]. However, the low frequency of animals receiving raw meat (only seven dogs) may have limited the statistical analysis, reducing the reliability of results related to this factor. Additionally, offering leftover food may indicate inadequate food preparation, storage, or disposal practices, potentially representing a shared risk, including possible exposure for humans [45].

Another finding was the higher prevalence of antibodies against *N. caninum* in dogs living in rural areas. Other studies report high seroprevalence in rural dogs and highlight the epidemiological importance of these infected animals, which promote environmental contamination and transmission of *N. caninum* to intermediate hosts such as cattle and wildlife [13, 14, 46], leading to economic losses [4] and impacts on biodiversity [34].

The results also demonstrated an association between *L. infantum* and *N. caninum* infections, suggesting that dogs infected with *L. infantum* are six times more likely to be coinfected with *N. caninum*, similarly to what has been reported in Italy [47]. In Brazil, *L. infantum* is the species responsible for canine visceral leishmaniasis [2], a multisystemic disease that leads to impairment of immune responses in dogs [48, 49]. Currently, there is no evidence demonstrating a direct relationship between these diseases, which have distinct transmission mechanisms. However, during *L. infantum* infection, the parasite may alter the immune response by infecting cells of the mononuclear phagocytic system, which act as antigen-presenting cells and activate CD4 + helper T (Th) lymphocytes [50]. This subset of immune cells participates in the adaptive cellular immune response and is directly associated with the ability of dogs to combat infections [51]. In this context, coinfection with *N. caninum* may exacerbate the severity of clinical signs [52].

One of the limitations of this study was the low frequency of dogs in some categories, such as the consumption of raw meat, which limited the statistical analysis, resulting in a type II error. In addition, information that could have enriched the analysis was not available, such as the direct contact of dogs with livestock.

## Conclusion

Infections by *T. gondii* and *N. caninum* are endemic in the municipalities of Barão de Melgaço and Nossa Senhora do Livramento in the Pantanal biome of Mato Grosso. The high prevalence of antibodies against *T. gondii* in dogs highlights their role as sentinels of the infection, indicating a potential risk to humans and wildlife. Regarding *N. caninum*, dogs as definitive hosts contribute to environmental contamination, promoting the maintenance of the parasite life cycle. Furthermore, dogs over 3 years of age, living together with other dogs and/or cats, and the absence of a public sewage system at the household were factors associated with the high seroprevalence observed for both agents. For *T. gondii*, infection was also associated to street access, report of rodents in the environment, and diet including leftover food, while *N. caninum* prevalence was higher in dogs from rural areas. Additionally, no association was found between coinfection by *T. gondii* and *N. caninum*, and dogs infected with *L. infantum* were not associated with the occurrence of antibodies against *T. gondii* but showed an increased risk of being infected with *N. caninum*.

## Data Availability

Data that support the findings described in this paper are available from the corresponding author upon reasonable request.
